# An Online Epilepsy Self-Management Tool for Patients With Epilepsy: A Formative Usability Study

**DOI:** 10.2196/79540

**Published:** 2026-08-05

**Authors:** Katarzyna Czerniak, Ross Shegog, Refugio Sepulveda, Robert Addy, Youngran Kim, Sahiti Myneni, Alejandra Garcia-Quintana, Kimberly Martin, David M Labiner

**Affiliations:** 1 Center for Health Promotion & Prevention Research, School of Public Health, The University of Texas Health Science Center at Houston, University Center Tower, Suite #2672-2, 7000 Fannin St., Houston, TX, 77030, United States, 1 713-500-9612; 2 Mel and Enid Zuckerman College of Public Health, University of Arizona, Tucson, AZ, United States; 3 Center for Health Care Data, School of Public Health, The University of Texas Health Science Center at Houston, Houston, TX, United States; 4 Department of Clinical and Health Informatics, D. Bradley McWilliams School of Biomedical Informatics, The University of Texas Health Science Center at Houston, Houston, TX, United States; 5 Epilepsy Foundation Central South Texas, San Antonio, TX, United States; 6 Department of Neurology, University of Arizona, Tucson, AZ, United States

**Keywords:** internet, mHealth, mobile health, digital health, eHealth, epilepsy, epilepsy self-management, usability

## Abstract

**Background:**

Epilepsy is a serious chronic neurological condition with no permanent cure. Continual self-management is important to mitigate seizure frequency and optimize quality of life in people with epilepsy who have greater disparities in accessing epilepsy care. The Management Information & Decision Support Epilepsy Tool (MINDSET [UTHealth, University of Arizona, and Radiant Digital]) was developed to enhance accessibility to epilepsy self-management (ESM) assessment and treatment. The purpose of this formative usability pilot study was to assess the user experience and functionality of MINDSET 2.0, an enhanced cross-platform online version of MINDSET, among a sample of patients with epilepsy prior to feasibility testing within neurology clinic settings.

**Methods:**

MINDSET 2.0 comprised an updated cross-platform architecture for easier accessibility and added quality of life, cognitive function, and social determinants assessments. User experience and functionality were assessed in January 2022. Six patients with epilepsy in Texas (n=4) and Arizona (n=2) participated in individual online usability sessions, completing a sociodemographic survey, accessing all components of MINDSET, and then completing usability rating scales and an exit interview. Logical inconsistencies in embedded algorithms were examined for the usability sample and in user case challenges to ensure functional fidelity.

**Results:**

Patients reported low adherence to ESM behaviors in each of the management domains. More than 80% of patients agreed that MINDSET 2.0 was acceptable, easy to use, likable, credible, of appropriate duration, and motivationally appealing. Patients agreed that the program helped them think about and manage their epilepsy more carefully, and that it improved decision-making between them and their health care providers (100%). Patients provided lower ratings (≤50%) and reported the greatest number of difficulties with their understanding of how to select goals and strategies, and develop an action plan due to constraints of item response options leading to user confusion. An inconsistency in algorithm branched logic was identified that related to translating depression scores into recommendations for depression self-management programming.

**Conclusions:**

The results replicated usability findings from earlier versions of MINDSET but also catalyzed adjustments to user survey response options and algorithm repair. The value of the formative user experience functionality assessment was demonstrated to ensure a high-fidelity program prior to feasibility testing in neurology clinic settings.

## Introduction

Epilepsy is a neurological condition, defined by repeated seizures of varied onset and type that affects more than 50 million people globally [1]. Approximately 2.9 million adults in the United States have active epilepsy [2,3]. People with epilepsy exhibit seizures that can manifest with temporary loss of awareness or consciousness; disturbed movement; altered vision, hearing, and taste; reduced mental function; anxiety and depression; and lower quality of life [4,5]. Epilepsy self-management (ESM) is important to enhance the self-efficacy of people with epilepsy in managing seizures and to optimize their quality of life [6]. ESM comprises a range of behaviors that include (1) medication management (eg, adherence to prescribed medication and clinical visit regimens), (2) seizure management (eg, preparation for, and response to, seizure episodes), (3) lifestyle management (eg, altering behaviors to avoid seizures and/or adverse consequences of seizures), and shared decision-making between the patient and their health care provider (HCP) [7,8]. ESM is associated with increased management self-efficacy and decision-making by people with epilepsy and their families, fewer breakthrough seizures, lower rates of hospitalization, and decreased risk for injury and premature death [8].

The COVID-19 pandemic disrupted traditional in-person clinic visits due to social distancing requirements and promoted digital solutions for patient treatment, such as telemedicine [9]. Digital decision support can assist HCPs to tailor patient care and increase patient awareness, knowledge, skills, and reach [10]. The Management Information & Decision Support Epilepsy Tool (MINDSET) was developed in 2015 to enhance patients with epilepsy and HCP assessment of ESM and has demonstrated improvement in adherence of patients with epilepsy to ESM [11-14]. An enhanced cross-platform web-based version of MINDSET, MINDSET 2.0, has been developed to enable improved function, phone-based accessibility, and added assessment and feedback on patient quality of life, cognitive difficulties, and social determinants of health [15-17]. User experience testing is a necessary antecedent of successful implementation of digital decision support to ensure it meets the specific needs of the priority population prior to deployment in the field [18]. The purpose of this study was to assess the user experience and functionality of MINDSET 2.0 with a sample of patients with epilepsy prior to deployment for feasibility testing within neurology clinic settings.

## Methods

### The Intervention

**Figure 1.**
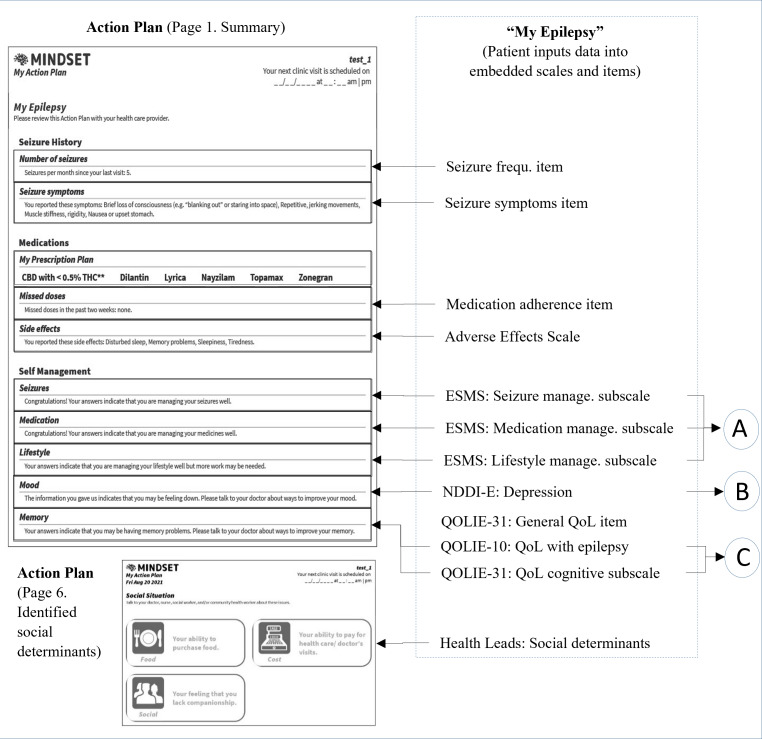
Decision logic for action plan tailoring part 1 (pages 1 and 6). ESMS: Epilepsy Self-management Survey; NDDI-E: Neurological Disorders Depression Inventory in Epilepsy; QoL: Quality of Life; QOLIE-10: Quality of Life in Epilepsy Inventory-10; QOLIE-31: Quality of Life in Epilepsy Inventory-31.

**Figure 2.**
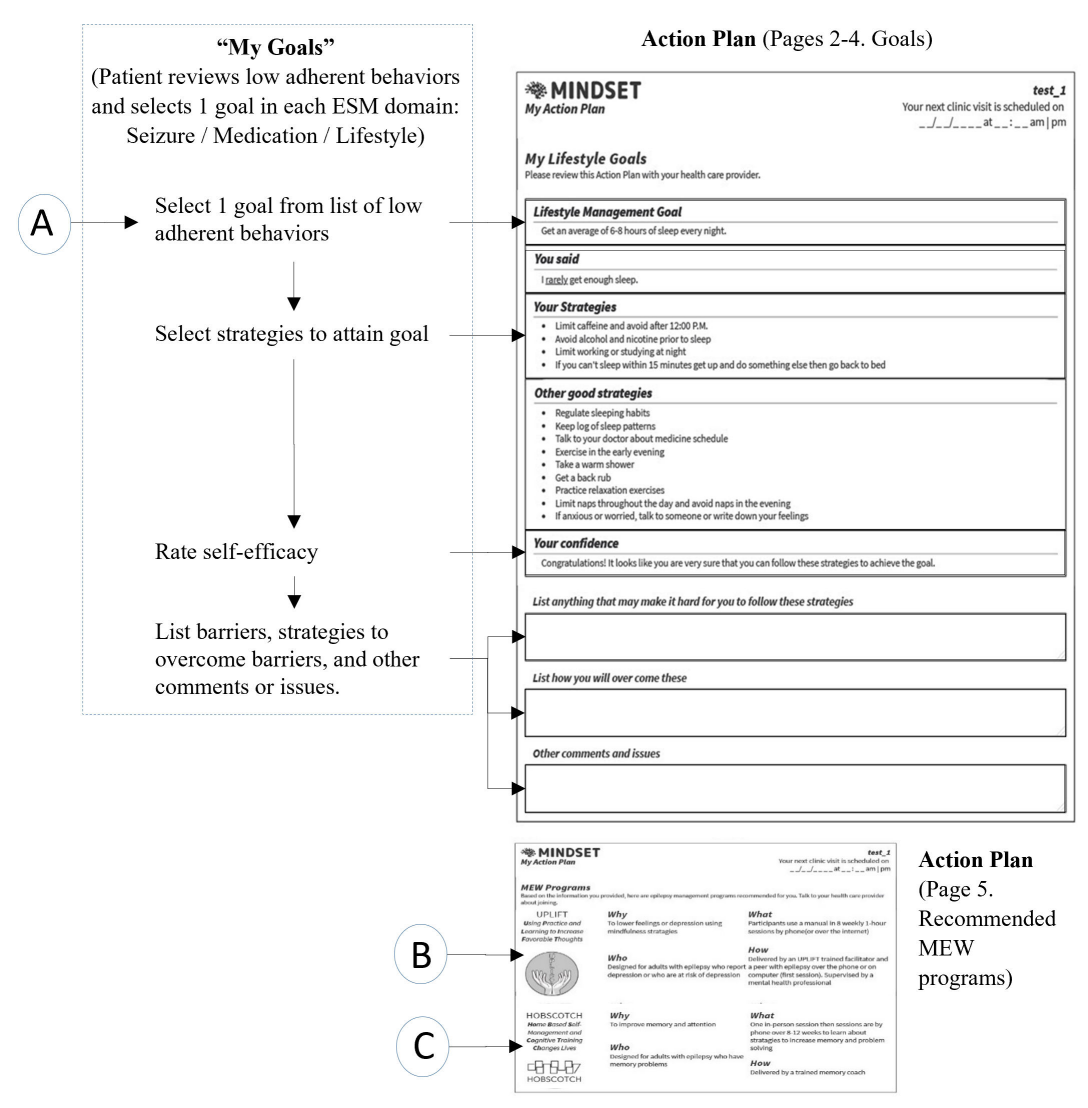
Decision logic for action plan part 2 (pages 2-5). ESM: epilepsy self-management; MEW: Managing Epilepsy Well.

MINDSET is a theoretically and empirically based digital ESM program, originally accessible on desktop or tablet computers in the clinic [11,19]. Patients choose their preferred language (English or Spanish) and input data (“My Epilepsy”) about their epilepsy condition (overall subjective health rating, seizure frequency in the last 30 days, seizure symptoms, antiseizure drug [ASD] prescription, and days of missed doses in the last 2 weeks) and their adherence to ESM behaviors within each of the 3 ESM domains (seizure, medication, and lifestyle management) using the Epilepsy Self-management Survey (ESMS) [20-22]. They complete the Neurological Disorders Depression Inventory in Epilepsy (NDDI-E) to assess depression [23] and the Liverpool Adverse Events Scale to assess ASD side effects [24-26] (Table 1). Patients select up to 3 ESM goals (one each for seizure, medication, and lifestyle management) to improve low adherent ESM behaviors (“My Goals”), choose strategies to accomplish these goals, rate their self-efficacy to achieve their selected goals, and list barriers to meet these goals [20,22]. The goals are designed to increase patient adherence to low adherent behaviors. A tailored goal-based action plan (AP; “My Action Plan”) is dynamically generated and is printed to review and discuss with the HCP during the clinic encounter (Figures 1 and 2). Page 1 of the AP comprises a summary of the patient’s epilepsy history and current condition, assessment of ESM behaviors and depression (Figure 2). Pages 2 to 4 summarize the selected goal, strategies, self-efficacy, and barriers for seizure, medication, and lifestyle management, respectively.

**Table 1.** Measures in MINDSET^a^ and user experience testing.

	Description
MINDSET measures (original version)	
Behavior	
Epilepsy Self-Management Scale^b^	A 38-item survey with 5-point Likert scale response format assessing frequency of epilepsy self-management practice in 5 management domains: seizure, medication, lifestyle, safety, and information (“I plan ahead so that I do not run out of seizure medication,” “I do things I enjoy to help manage stress,” etc). Each item is rated from 1 (never) to 5 (always) with negative behaviors reverse scored. Cronbach α: 0.81‐0.84 [20,27-30].
Neurological Disorders Depression Inventory for Epilepsy	A 6-item survey with 4-point Likert response format assessing frequency of depressive symptoms within the last 2 weeks (range is 1 “never” to 4 “always or often”). Final score is sum of all responses (range 6‐24). Scores ≥15 indicate depressive symptoms (specificity: 90%, sensitivity: 81%, and positive predictive value of 0.62 for detecting major depressive disorder [23]). Cronbach α: 0.85, test-retest reliability: 0.78 [31,32].
Clinical outcomes	
Seizure frequency	Frequency of seizures experienced in the past 4 weeks. People with epilepsy input a number in an open textbox format.
Adverse Drug Effects Scale^c^	Assesses medication side effects experienced during the previous 4 weeks from a list of 19 adverse effects. The original uses a 4-point Likert scale response set: 1 (never a problem) to 4 (always a problem). Score range: 19 to 76. Cronbach α: 0.90 (baseline) and 0.91 (3-month follow-up) [24-26].
Missed doses item	Assesses how many doses of anti-seizure medications patients missed in the last 2 weeks. People with epilepsy can select any number between 1 and 20 as well as nonnumeric answers which are “more than 20,” “don’t know,” and “Not currently on any seizure medications.”
MINDSET 2.0 measures (enhanced version): MINDSET 2.0 contains all the measures from the original version listed above plus the additional measures listed below	
Behavior	
QOLIE-10^d^ Survey^e^	Assesses how often epilepsy has affected the patient’s health and daily activities in the last 4 weeks. Scale contains 10 epilepsy-specific items from 3 domains: epilepsy effects (memory, physical, and mental effects of medication), mental health (energy, depression, and general quality of life), and role functioning (seizure worry, work, driving, and social limits). Likert responses vary and include: 1 (all of the time) to 6 (none of the time), 1 (a great deal) to 5 (not at all), and 1 (not at all bothersome) to 5 (extremely bothersome). Test-retest reliability and internal consistency are high [33].
QOLIE-31^f^ Survey (single item)^g^	A single item that assesses overall perceived quality of life using a rating scale of 0 (worst possible quality of life) to 10 (best possible quality of life). This item is analogous to a general health question that often “front ends” a clinic visit enquiry. A score is calculated by converting the raw numeric values to 0‐100 point scores. Higher scores reflect better overall quality of life [34].
QOLIE-31 cognitive subscale	This 6-item self-report subscale assesses how often patients experience epilepsy-related cognitive issues in their daily lives. Response selections vary by item and include 1 (all of the time) to 6 (none of the time), 1 (a great deal) to 4 (not at all), and 1 (not at all bothersome) to 5 (extremely bothersome). A score is calculated by converting the raw numeric values to 0‐100 point scores with higher scores indicating better cognitive health [34].
Health Leads social determinants inventory^h^	An 8-item social determinants inventory screening tool to assess if patients are having difficulties in 8 areas of their daily lives. People with epilepsy respond Yes or No to each of the 8 items [35].
User experience testing measures	
Demographic survey	A 26-item demographics survey that collects information about gender, age, race, ethnicity, residency in the United States, education, marital status, income, employment status, health care status, seizure history, quality of life, computer and smartphone access, and language preference [36].
MINDSET Usability Rating Scale^i^	An adapted 36-item rating inventory to assess usability parameters of acceptability, likability, credibility, motivational appeal, the content, duration to complete the program, usefulness, perceived impact on epilepsy, ease of use, and understandability. Seven items were added to cover new content in MINDSET 2.0. This rating inventory assessed specific functions in MINDSET and enabled comparison with results from previous MINDSET studies [12,37].
Feasibility exit interview^j^	A 30-minute structured exit interview conducted after survey data was collected. A trained facilitator asked 6 questions, with additional prompts as needed, to assess the patient’s experience and their perceptions of the strengths, weaknesses, utility, and appeal of MINDSET 2.0, and to elicit recommendations for enhancement [12,37].

a MINDSET: Management Information & Decision Support Epilepsy Tool.

b MINDSET patients are considered adherent if the positive behaviors occur “usually” or “always.” Epilepsy self-management behaviors are flagged by the program as nonadherent if patients report “never,” “rarely,” or “sometimes” for a positive behavior. This is reversed for negative behaviors.

c In MINDSET, the response set was adjusted to a check box selection of any side effects and 3 additional adverse effects were included informed by neurologist review. All reported medication side effects are listed on the action plan produced at the end of the program, which helps health care providers identify patients needing treatment adjustments during clinic visits. Adverse drug effects choices: unsteadiness, tiredness, restlessness, aggression, nervousness, hair loss, skin changes or rash, blurred vision, upset stomach, concentration difficulty, mouth and gum problems, shaky hands, weight gain, dizziness, sleepiness, depression, memory problems, and disturbed sleep.

d QOLIE-10: Quality of Life in Epilepsy Inventory-10.

e Items with opposite response sets are reverse scored so that positive responses are lower numbers and negative responses higher. The total score is calculated by averaging the items answered. Total scores range between 0 and 6, with lower scores indicating better quality of life.

f QOLIE-31: Quality of Life in Epilepsy Inventory-31.

g The item is designed for assessing Quality of Life but doesn’t itself trigger intervention recommendations. All those who use MINDSET receive a recommendation to Program for Active Consumer Engagement in Self-Management regardless of their answer choices to ensure that everyone has access to more intense intervention should they one day need it.

h Health Leader assesses food insecurity, housing instability, financial resource strain, transportation challenges, social isolation, lack of childcare, healthcare illiteracy, and utility needs.

i The 36-item inventory provides statements (Table 2) about various aspects of MINDSET 2.0 and the action plan for which the patient provides a rating using binary response options including “yes/no,” “helpful/not helpful,” “can be trusted/can’t be trusted.” The patient also rates the structure and design (eg, colors, font size, and screen layout) using a 5-point Likert scale (dislike a lot (1) to like a lot (5)), and ease of use using 4-point Likert scales (not easy (1) to very easy (4)).

j Questions included: “Describe your overall experience with MINDSET 2.0; What did you like best or least about MINDSET 2.0 and why?; Was there anything that made you feel upset, embarrassed, or uncomfortable?; How relevant or relatable was the information presented to you?; What would you have liked to see but did not see?; What would you tell other patients about MINDSET 2.0?

**Figure 3.**
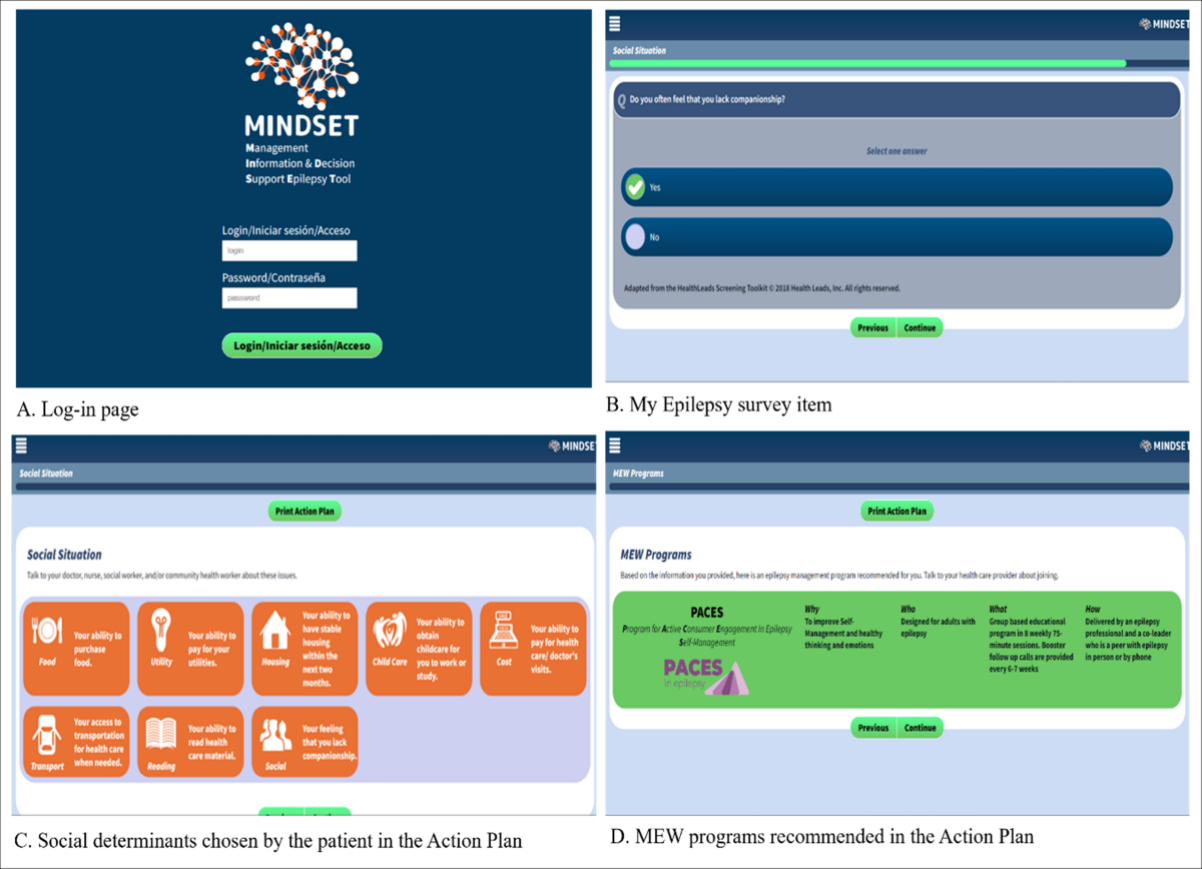
Screen captures of MINDSET 2.0. MEW: Managing Epilepsy Well.

MINDSET has been enhanced to MINDSET 2.0 with added features (Table 1) that include (1) accessibility on any device with an internet connection, (2) assessment of depression using the NDDI-E where a score of ≥15 triggers a summary message in the AP to discuss mood and a recommendation that the patient may benefit from the Using Practice and Learning to Increase Favorable Thoughts (UPLIFT) program designed to train patients with epilepsy to manage comorbid depression [38], and (3) assessment of quality of life using the Quality of Life in Epilepsy Inventory-10 (QOLIE-10) [33] and cognitive (memory) difficulties using the Quality of Life in Epilepsy Inventory-31 (QOLIE-31) cognitive subscale [34]. A QOLIE-31 cognitive subscale score of ≤70 and/or the QOLIE-10 memory item can trigger a recommendation that the patient may benefit from the Home-Based Self-Management and Cognitive Training Changes Lives (HOBSCOTCH) program designed to train patients with epilepsy to manage comorbid memory problems (Figure 2, AP page 5) [39]. Additional features included (4) a default recommendation for the Program for Active Consumer Engagement in Self-Management (PACES) as a foundational ESM training program given its suitability for all patients with epilepsy. UPLIFT, HOBSCOTCH, and PACES are available in English and Spanish. Lastly, MINDSET 2.0 added an (5) assessment of social determinants using the Health Leads social determinant inventory [35] to cue discussion about possible social service needs (Figure 3). The HCP can link the patient to recommended epilepsy programs and connect them with community resources as needed.

### User Experience Testing

In-house alpha testing by the research team was conducted to ensure that the content was accurate and consistent with current clinic practice, that spelling and grammar were error-free, that inputted data corresponded to the backend database, and that the Spanish translation was correct. User experience testing occurred in January 2022. A convenience sample of 7 patients with epilepsy was recruited from the Epilepsy Foundation Central & South Texas (EFCST) clinic in San Antonio, Texas (n=5) and the Banner Clinic in Tucson, Arizona (n=2). Inclusion criteria were that patients be 18 years or older; have a diagnosis of epilepsy; and have access to a computer, phone, or tablet with a working internet connection. MINDSET was designed for patients with epilepsy who are able to self-manage their epilepsy, so neurologists determined exclusion of patients with neurological, medical, motor disorders, or learning problems as well as psychiatric and/or behavioral problems that would excessively inhibit their ability to use MINDSET 2.0, complete surveys, or practice ESM activities.

Clinic staff contacted eligible patients via phone and introduced those who verbally consented to the research team coordinator who arranged a testing session. Sessions were conducted on Zoom (Zoom Communications, Inc), a familiar platform to most participants, due to clinical mandates for COVID-19–related social distancing. In each session, participants completed a consent form, a 26-item sociodemographic survey, accessed all components of MINDSET (eg, the screening tool, patient profile, recommendations, and AP), and then completed an adapted 36-item usability rating scale (Multimedia Appendix 1) and a 30-minute structured exit interview (Table 1). The research team coordinators guided participants through the session and noted any issues in a problem log. The coordinators comprised a doctoral candidate in Behavioral Sciences with 6 years of experience in qualitative research in Texas and an assistant research professor at a state college of public health with an adjunct appointment at a department of neurology in Arizona. A bilingual master’s in public health (MPH) student took notes and served as a Spanish translator for patients electing to complete MINDSET 2.0 and study surveys in Spanish. Participants were allowed to complete the study with assistance from caregivers if needed. Participants were compensated for their time with Amazon e-gift cards valued at US $60 at the end of the session. User experience data were assessed with descriptive statistics using Stata 18 (StataCorp LLC) [40]. A priori criteria user agreement of at least 70% was set as a usability benchmark. Features that fell below this were identified for modification. Exit interviews were recorded, and transcripts were assessed and collated to qualify each usability parameter.

### Ethical Considerations

The study was approved by the institutional review boards of the University of Texas Health Science Center at Houston (HSC-SPH-19‐0893) and the University of Arizona (2005674587). Informed consent was obtained from all participants who were given the option to withdraw from the study at any time. All data collected in the study were deidentified to maintain patient privacy and confidentiality.

### Functionality Testing

The functionality assessment comprised (1) noting “bugs” or suboptimal performance in a program log and (2) reviewing algorithm logic fidelity from the user experience testing data, whereby inputted participant data were compared to AP output and assessed against an a priori benchmark for success of 100% correspondence. Staff manually calculated participant scores (eg, NDDI-E depression scores and QOLIE-31 cognitive scores) to predict expected recommendations and compared these to the actual recommendations generated on the AP to test functionality of the algorithms. The assessment also included (3) challenging the MINDSET 2.0 algorithm function with an array of user profile test cases ranging from a “worst case” ESM patient profile to a “best case” and variations between these. A “worst case” patient answered all questions negatively and a “best case” positively. User test cases were assigned unique ESM behavior profiles for seizure, medication, and lifestyle management and varied depression, Quality of Life (QoL), and social determinant indices. Each tester was assigned a predetermined set of responses to ensure that all item answers were inputted at least once. Testers selected the corresponding goal for each assigned at-risk item in the profile to ensure all goals were selected at least once. APs were then reviewed to determine that messages and goals were logically consistent with the data input. Similar to the user experience algorithm review, an a priori benchmark for success was set at 100% correspondence between data input and AP output.

## Results

### Participant Demographics, Epilepsy, and Self-Management Characteristics

Six participants completed MINDSET. One participant withdrew without completing the AP citing epilepsy cognitive impairment. Participants had a mean age of 37.3 (SD 15) years, were equally male and female (n=3, 50%), Hispanic or Latino (n=6, 100%), with a mean US residency of 23 (SD 13.2) years, mainly with high school (n=3, 50%) or 1‐3 years of college (n=2, 33.3%) education, single (n=4, 66.7%), with income below US $50K (n=5, 83.3%), and were either health insured (n=3, 50%) or not health insured (n=3, 50%; Table 2). Most reported having access to a smartphone and/or a computer (n=5, ≥83.3%) and preferred English or mainly English for reading, speaking at home, and thinking (n=5, 83.3%).

**Table 2.** Participant self-reported demographic characteristics (N=6).

Construct		Value
Age (years), mean (SD); median (IQR); range		37.3 (15); 39.5 (21-50); 19-55
Sex, n (%)		
Female		3 (50.0)
Male		3 (50.0)
Ethnicity		
Hispanic		6 (100)
Race		
White		6 (100)
Residency in United States (years), mean (SD); median (IQR); range		23.7 (13.2); 20 (13-35); 10‐44
Education level, n (%)		
GED^a^ (HS^b^ graduate)		3 (50.0)
College 1‐3 years		2 (33.3)
College ≥4 years		1 (16.7)
Marital status, n (%)		
Single		4 (66.7)
Married or living with other		2 (33.3)
Income level (US $), n (%)		
< $10,000		2 (33.3)
$10,000‐$49,000		3 (50.0)
≥$50,000		1 (16.7)
Employment status, n (%)		
Employed for wages		2 (33.3)
Not working (unable, no work, and student)		4 (66.7)
Health care insurance, n (%)		
Employer		1 (16.7)
Public		1 (16.7)
Medicaid		1 (16.7)
No coverage		3 (50.0)
Technology access, n (%)		
Computer		5 (83.3)
Smartphone		6 (100)
Language preferred with friends, n (%)		
English		4 (66.7)
English >Spanish		1 (16.7)
Language preferred (speaking at home), n (%)		
English		3 (50.0)
English >Spanish		2 (33.4)
Spanish		1 (16.7)
Language preference (thinking), n (%)		
English		5 (83.3)
English >Spanish		1 (16.7)

a GED: General Educational Development.

b HS: high school.

Participants rated their QoL as mostly “very good” (n=3, 50%; Table 3). Their first ever seizure occurred between ages 2 and 15 years. They had active epilepsy, reporting seizures in the last 12 months (n=6), and in the last 30 days (n=3). They mostly reported 5 to 6 seizures in the last month and 4 seizures since their last clinic visit. They reported adherence to their ASD dosing schedule, on average missing a dose on one day in the last 2 weeks. They reported 3 to 8 side effects. The sample mean NDDI-E score was 12.8 (SD 5.6) with 2 participants scoring ≥15. The mean QOLIE-31 item score was 85 (SD 15.2) and the mean QOLIE-31 cognitive subscale score was 51.6 (SD 33.3), indicating poor QoL. However, the mean QOLIE-10 score was 2.5 (SD 1.02), indicating good QoL in relation to patients’ epilepsy. The greatest adherence to ESM was for medicine management behaviors (mean 4.5, SD 0.70), and the lowest adherence was for lifestyle management behaviors (mean 3.9, SD 0.41) and information management behaviors (mean 3.5, SD 0.63; Table 3).

**Table 3.** Participant self-reported clinical characteristics (N=6).

Construct and category	Value
Quality of Life^a^	
Quality of Life (last 30 days)	
Excellent	1 (16.7)
Very good	3 (50.0)
Fair	2 (33.3)
Epilepsy duration (years), mean (SD); median (IQR); range	
Age at first seizure	8.5 (5.3); 7.5 (5‐14); 2‐15
Age at diagnosis	9.8 (6.8); 8.5 (5‐15); 2‐20
Seizures^a^	
Seizure frequency (yes or no)	
Last 12 months	6 (100)
Last 30 days	3 (50)
Seizure frequency (number of seizures in 30 days), mean (SD); median (IQR); range	13.3 (14.4); 5 (5-30); 5‐30
Seizures^b^, mean (SD); median (IQR); range	
Seizure frequency since last clinic visit	6.3 (7.6); 4 (0‐10); 0‐20
Medication, mean (SD); median (IQR); range	
Number of days in the last 2 weeks ASD^c^ medications missed	0.4 (0.54); 0 (0‐1); 0‐1
Number of ASD side effects (last 2 wk)	5.7 (0.3); 6.5 (3-8); 1‐9
Depression, mean (SD); median (IQR); range; number of participants with depression (score ≥15)	
Mood (NDDI-E^d^)^e^	12.8 (5.7); 12 (8-19); 6‐20; 2
Cognitive problems, mean (SD); median (IQR); range	
QOLIE-31 cognitive subscale^f^	51.6 (33.3); 47.3 (22.2‐79.4); 13.3‐100
Quality of Life^b^, mean score (SD); median (IQR); range	
QOLIE-31^g^	85 (15.2); 85 (80-100); 60‐100
QOLIE-10^h^	2.5 (1.02); 2.3 (1.7‐3); 1.5‐4.3
Behavior	
Epilepsy self-management^i^, mean score (SD); median (IQR)	
Seizure	4.4 (0.60); 4.6 (4-5)
Medicine	4.5 (0.70); 4.7 (4.3‐5)
Lifestyle	3.9 (0.41); 3.8 (3.6‐4.2)
Safety	4.2 (0.73); 4.1 (3.8‐5)
Information	3.5 (0.63); 3.41(3-4)

a Collected using the demographics survey.

b Collected using the data collection portion of the MINDSET program.

c ASD: antiseizure drug.

d NDDI-E: Neurological Disorders Depression Inventory in Epilepsy.

e Responses, Likert scale—never: 1 to always or often: 4; symptoms, maximum score 24, score ≥15 is symptomatic.

f Cognitive subscale (items 12, 15, 16, 17, 18, and 26) from QOLIE-31 scale. Combined score of ≥70 is obtained if the memory problem is “little” or “none” of the time.

g QoLIE-31 item 1. Higher scores reflect better quality of life; lower ones, worse quality of life. Possible range is 0-100.

h QOLIE-10 scale. Total scores range between 0 and 6, with lower scores indicating better quality of life.

i Self-management range of mean item scores from a 5-point Likert response set with 1-Never, 2-Rarely, 3-Sometimes, 4-Most of the time, 5-Always

### User Experience

Participants took approximately 30‐60 minutes to complete MINDSET, up to 30 minutes to complete the survey and ratings scale, and up to 30 minutes to complete the exit interview. Participants mostly selected the English language version (n=5, 83.3%). More than 80% of participants agreed that MINDSET 2.0 was acceptable, likable, and motivationally appealing (that they would use it again during future visits and recommend it to others) and indicated it to be “exciting,” “colorful,” and “relevant” (Table 4). They rated it as credible (n=6, 100%) and of appropriate duration (n=5, 83.3%), and that, in its entirety, it was “easy to use” and “simple” (n=6, 100%), and that the words used were understandable (n=6, 100%), requiring no help to answer survey items (n=5, 83.3%). They commented favorably on its comprehensiveness, stating that it was “thorough” and “in-depth,” and appreciated the degree of detail in the tailored AP. They rated it as providing needed (n=5, 83.3%) and helpful (n=6, 100%) information, recommending ESM programs appropriate to their needs (n=5, 83.3%), and that it helped them think about and manage their epilepsy more carefully, and that it would improve decision-making with their HCPs (n=6, 100%). They rated it as more useful than other programs they had used in the clinic (n=5, 83.3%) and described it as an effective and “innovative” evaluation tool that introduced them to new things and assisted them to understand broader lifestyle factors associated with seizure reduction such as sleep, stress, diet, and substance use. Participant recommendations for enhancement of MINDSET content were confined to suggestions that MINDSET “assess family dynamics” due to the reliance of many patients with epilepsy on family members to assist with seizure and medication management, and provide support to mitigate stigma and financial strain [7,13]. Requests were also made that MINDSET 2.0 flag suicidal ideation based on participant experience.

**Table 4.** Patient agreement on usability parameters.

Usability parameters and items		Agreement, n (%)
Acceptability		
Entire program		6 (100)
Likeability^a^ (interface design)		
Colors used in this program		5 (83.3)
Buttons and sliders used in this program		6 (100)
Size of the letters		5 (83.3)
Device used		5 (83.3)
Layout of the screen		6 (100)
Motivational appeal		
I would recommend MS+ to other patients: Yes		6 (100)
I would use MS+ again in a clinic visit: Yes		6 (100)
Credibility		
I think the information I got from MS+ can be trusted		6 (100)
Duration		
Time to use was		5 (83.3)
Just right “Too Long”		1 (16.7)
Usefulness		
I think the information I got from MS+ was helpful		6 (100)
MS+ gave me most of the information that I needed		6 (83.3)
The MEW^b^ Network programs that MS+ suggested is appropriate for my needs		5 (83.3)
Having MS+ suggest MEW^b^ Network programs was helpful		5 (83.3)
MS+ is as useful or more useful than other seizure programs that I have used^c^		1 (100)
Perceived impact		
I think the information provided...:		
Helped me to think carefully about my epilepsy		6 (100)
Will help me talk to my doctor or nurse about my epilepsy		6 (100)
Will help me manage my epilepsy better		6 (100)
Easy to use		
It was easy to^d^: Enter your responses into MS		6 (100)
I think MS was easy to uses		6 (100)
Understandability		
I did not need help to answer questions		5 (83.3)
I knew and understood most of the words		6 (100)
Action plan development online		
Understand instructions for “My Goals”		0 (0)
Understand the action plan		2 (33.3)
It was easy to^d^ choose a goal to work on		3 (50)
Choose your strategies		3 (50)
Action plan development		
Understanding the PDF version of the action plan		3 (50)
Understanding the PDF action plan layout		0 (0)
Understanding the PDF action plan content		2 (33.3)
Downloading PDF version of action plan		1 (16.7)

a Likert scale: like a lot (1)–dislike a lot (4); Like = “Likes a lot” and “like.”

b MEW: Managing Epilepsy Well.

c Respondents who have not used another program for comparison (n=5).

d Likert scale: very easy–not easy; easy = ”somewhat easy” and “very easy.”

### Item Formatting

Patients reported lower agreement with the ease of selecting goals and strategies (n=3, 50%) and understanding and using the AP (n=2, ≤33%). The MINDSET 2.0 AP occasionally provided feedback and goal options that seemed to the participants to be misaligned or incongruous to their daily ESM. Participants expressed some frustration and confusion as to why certain behaviors seemed incorrectly flagged as low adherent. The interviews revealed that many patients had difficulty providing responses to the questions due to a restricted 4-point Likert scale. For example, to the item “I use safety precautions in the kitchen,” a patient in Texas responded “never,” because she is “never in the kitchen.” Given this rationale, the more accurate response would have been “not applicable” rather than a response indicative of nonadherence. A patient in Arizona responded, “all the time” to the statement “I go swimming alone,” their rationale being that they do not need to “hold on to anyone or anything” when going to swim rather than being unaccompanied. Patients’ misinterpretation of the meaning and purpose of the questions suggested problems with understandability at the item level. Despite published findings of empirically sound psychometrics for the ESM survey, this feedback indicated the need to incorporate “not applicable” response options into items to increase response validity. In interviews, most reported difficulties were in the development and understanding of the AP. One participant reported that it seemed “repetitive” and “arduous,” while another expressed difficulty in understanding how to use it, inclusive of selecting goals and strategies, interpreting the output, and AP download.

### Patient Profiling and Goal Selection

MINDSET 2.0 exhibited no connectivity or bandwidth lags or data dropout, and back-end data collection was error-free. ESM was positively skewed and highest for medicine, seizure, and safety management subdomains (Table 3). The number of low adherence behaviors per participant ranged from 1 or 2 for seizure management, 2 to 6 for medication management, and 2 to 15 for lifestyle management (Table 5). Most patients (n=4, 66.7%) reported high adherence to medicine management behaviors, which was compatible with the low frequency of missed ASDs. Selected goals were “always keeping enough medicine” to avoid running out and “using a pill container or pill box” for ASDs (Table 5, column 2). All patients were flagged as having at least one low adherent lifestyle behavior. Selected goals focused on stress and relaxation techniques, getting an average of 6‐8 hours of sleep (n=2), carrying an epilepsy ID, forming social connections, and eating a healthy diet. The goals were unique to each participant apart from “getting enough sleep,” which was selected by 2 patients. For any chosen goal, patients selected between 0 and 7 strategies (Table 5, Column 3). The majority selected one strategy from the list of strategies provided. Two patients listed potential barriers to meeting their goals, both for seizure management, that related to the need to get a primary care doctor or neurologist, and health insurance (Table 5, column 3). No participant self-efficacy ratings were downloaded in the AP. This was traced to an error in coding logic failure that was converting categorical Likert scale scores into binary scores.

**Table 5.** Flagged ESM^a^ behaviors, participant-selected goals, and strategies^b^.

Specific participants	Low adherent (flagged) ESM behaviors^c^		Goal^d^	Strategies^e^ and barriers^f^
Seizure management (I Rarely...)^g^			(Always...)	
1	Call my doctor if I am having more seizures than usual.		Contact your doctor or nurse when you are experiencing increased seizures.	Other [“Applying for health insurance to have a primary doctor”] Barrier [“Don’t have primary doctor or health insurance”]
2	Call my doctor if I am having more seizures than usual. Have blood tests done when the doctor orders them.		Same as above.	Keep a log of seizures Barrier [“…to find a neuro that I can work with…”]
6	Call my doctor if I am having more seizures than usual.		Same as above.	None [“Memorizando”]
Medication management (I Rarely...)^h^			(Always...)	
2	Spread out the time between doses when my seizure medication is running out Plan ahead so that I do not run out of my seizure medicine Take my seizure medicine at the same time each day Use a pill container or pill box for my seizure medicines Have a way to remind myself to take my seizure medicine Forget to take my seizure medicine^i^		Keep enough medicine.	Ask for a refill of medicines at least 2 weeks before If away from home for a period of time, make a plan so that you don’t run out of medicine
4	Plan ahead so that I do not run out of my seizure medicine Use a pill container or pill box for my seizure medicines		Use a pill container or pill box.	Use a pill box
Lifestyle management (I Rarely...)			(Always...)	
1	Have ways to help relax to reduce chances of having a seizure Eat regular, healthy meals Keep a record of seizures Talk to or chat online with other people who have epilepsy Stay out late at night^i^ Climb on high stools or chairs or ladders^i^		Reduce your stress and practice relaxation techniques.	Prioritize what is important in your life
2	Make sure I get enough sleep Do things that I enjoy to help manage stress Eat regular, healthy meals Keep a record of seizures Use safety precautions when in the kitchen Keep track of the side effects of seizure medicine Check with my doctor before taking other medicines. Go swimming alone^i^ Climb on high stools or chairs or ladders^i^ Get enough exercise Avoid driving until seizure free for the minimum amount of time required in my state Wear a bracelet or necklace, or carry information stating that I have seizures/epilepsy Tell doctor or nurse when I think I’m having unexpected side effects from seizure meds Use power tools such as electric saws, electric hedge trimmers, or electric knives without an automatic shutoff^i^		Get an average of 6‐8 hours of sleep every night.	Regulate sleeping habits Keep log of sleep patterns Exercise in the early evening Practice relaxation exercises
3	Do things that I enjoy to help manage stress Wear a bracelet or necklace, or carry information stating that I have seizures or epilepsy Carry a cell phone to call someone if I need help Use safety precautions when in the kitchen Keep a record of seizures Keep track of side effects of seizure meds		Always carry an ID stating your epilepsy.	None selected
4	Make sure I get enough sleep Get enough exercise Wear a bracelet or necklace, or carry information stating that I have seizures or epilepsy Talk to or chat online with other people who have epilepsy Stay out late at night^i^ Climb on high stools or chairs or ladders^i^		Get an average of 6‐8 hours of sleep every night.	Regulate sleeping habits Keep log of sleep patterns Limit working or studying at night Take a warm shower Limit naps throughout day and avoid naps in the evening If anxious or worried, talk to someone or write down feelings If can’t sleep within 15 minutes get up and do something else then go back to bed.
5	Avoid driving until seizure free for the minimum amount of time required in my state Talk to or chat online with other people who have epilepsy		Find people to talk to or chat online with who also have epilepsy.	Join an epilepsy support group (local or community groups or online)
6	Have ways to help relax to reduce chances of having a seizure Avoid driving until seizure free for the minimum amount of time required in my state Keep track of side effects of seizure meds Talk to or chat online with other people who have epilepsy Get enough exercise Keep a record of seizures Keep the water temperature in my home low enough so that it would not burn me if I had a seizure in the shower Wear a bracelet or necklace, or carry information stating that I have seizures or epilepsy Do things that I enjoy to help manage stress Eat regular, healthy meals		Take control of your diet.	Eat a well-balanced diet

a ESM: epilepsy self-management.

b Self-efficacy was not collected because the Likert scale response was converted to a binary response. This was repaired subsequent to this study.

c Low adherent epilepsy self-management behaviors are defined as having a frequency of “never,” “rarely,” or “sometimes” on a 5-point Likert scale.

d Selected by the participant.

e When an “other” category was selected, the patient could provide a written response in the text box provided.

f Patients have the option to list barriers that impede their ability to meet goals as well as list strategies used to overcome barriers in an open-ended data entry box if desired.

g Participants 3, 4, and 5 had no flagged behaviors.

h Participants 1, 3, 5, and 6 had no flagged behaviors.

i For negative behaviors, answer selections and scores were reversed.

### ESM Program Recommendation Logic

All participants reported having no past use or experience with any of the Managing Epilepsy Well (MEW) Network programs (UPLIFT, HOBSCOTCH, or PACES). In accordance with the MINDSET 2.0 design, all patients were recommended to attend the PACES epilepsy training program, which was the default recommendation. The HOBSCOTCH cognitive skills training program recommendations for patients flagged as having cognitive difficulties (n=5) were also consistent with the MINDSET logic model (Table 6). However, correspondence between predicted and actual recommendations for the UPLIFT program was less than 100% for 2 participants (Table 6, participants #3 and #6). These 2 participants were flagged as having depressive symptoms (NDDI-E score >15) but were not recommended by MINDSET 2.0 to attend the UPLIFT depression program despite no prior history with this program. This represented a failure of the algorithm to translate NDDI-E depression scores into a recommendation to remediate for depression and was traced to incorrect algorithm decision logic.

**Table 6.** ESM^a^ program recommendation algorithm functionality.

Data input (“my epilepsy” survey)										Data output (action plan)						Correspondence of predicted to actual (%)^b^
Participant^c^	ESM Behavior Flagged^d^			Depression^e^	Memory^f^		Previous Exposure to P/U/H^g^			MEW^h^ Program Recommendation^i^						
	S	M	L	NDDI-E^j^	QOLIE-31^k^ cognitive scale	QOLIE-10^l^ item	P	U	H	PACES^m^		UPLIFT^n^		HOB^o^		
										Pred.	AP^p^	Pred.	AP	Pred.	AP	
1	✓	—	✓	—	—	✓	No	No	No	✓	✓	—	—	✓	✓	100
2	✓	✓	✓	—	✓	✓	No	No	No	✓	✓	—	—	✓	✓	100
3	—	—	✓	✓	✓	✓	No	No	No	✓	✓	✓	—	✓	✓	66.6^q^
4	—	✓	✓	—	✓	✓	No	No	No	✓	✓	—	—	✓	✓	100
5	—	—	✓	—	—	—	No	No	No	✓	✓	—	—	—	—	100
6	✓	—	✓	✓	✓	✓	No	No	No	✓	✓	✓	—	✓	✓	66.6^q^

a ESM: epilepsy self-management.

b Correspondence of predicted to actual recommendations. The benchmark is 100%.

c Represents a specific patient with epilepsy participant.

d ESM domains (S=Seizure; M=Medicine; L=Lifestyle). A domain is flagged with a checkmark if a behavior within that domain has low adherence. Goal options are generated for each low adherent behavior for the patient to select during the action plan development

e Depression is indicated on the action plan if a patient obtains a summed score of 15 or more on the Neurological Disorders Depression Inventory in Epilepsy scale.

f Cognitive issues are indicated on the action plan if a patient obtains a score of 70% or lower on the Quality of Life in Epilepsy Inventory-31 cognitive subscale OR answers 3, 4, or 5 (extremely bothersome) on the Quality of Life in Epilepsy Inventory-10 item about memory OR both.

g Past exposure to Managing Epilepsy Well Network programs (P=PACES; U=UPLIFT; H=HOBSCOTCH) is a criterion contributing to determine if a patient receives a recommendation to one or more of these programs. Patients self-report any past exposure as Yes (Y), No (N), or Don’t Know (DK) for each of the 3 Managing Epilepsy Well programs.

h MEW: Managing Epilepsy Well.

i Predicted (pred.) and actual (AP) recommendations for Managing Epilepsy Well Network programs are shown (✓=recommended; - =not recommended).

j NDDI-E: Neurological Disorders Depression Inventory in Epilepsy.

k QOLIE-31: Quality of Life in Epilepsy Inventory-31.

l QOLIE-10: Quality of Life in Epilepsy Inventory-10.

m PACES: Program for Active Consumer Engagement in Self-Management

n UPLIFT: Using Practice and Learning to Increase Favorable Thoughts.

o HOBSCOTCH: Home-Based Self-Management and Cognitive Training Changes Lives.

p AP: action plan.

q Participants #3 and #6 had noncorresponding results where depression scores should have caused a recommendation for UPLIFT in the action plan.

### Social Determinant Feedback

Four patients reported at least one social determinant that may have hindered their epilepsy management, mainly food insecurity (n=3) and financial resource strain (n=3). Other listed determinants were housing instability (n=2), low health literacy (n=2), transportation challenges (n=1), utility needs (n=1), and social isolation (n=1).

## Discussion

### Principal Findings

The purpose of this study was to assess the usability of MINDSET 2.0 prior to deployment for feasibility testing in neurology clinics that serve patients with epilepsy. The 6 testers, who were people with a doctor’s diagnosis of epilepsy, were experienced in epilepsy management and forthcoming about recommending improvements to MINDSET 2.0 to enhance its comprehensiveness and relevance. Consistent with the health disparities often cited for patients with epilepsy at the national level, the participants reported limited insurance coverage, education, and employment, and the most reported social determinants were food insecurity and financial strain that can impede their ability to manage their epilepsy. The usability findings were largely consistent with previous studies of MINDSET in that the patients agreed that MINDSET 2.0 was acceptable, likable, credible, easy to use, and useful for decision-making and ESM, meeting a priori criteria of 70% agreement [12,37]. Patients reported less overall adherence to, and competency in, information management (monitoring and recording seizures and symptoms, and communicating about epilepsy) and lifestyle management (eg, ensuring adequate exercise, sleep, and strategies to relieve stress) compared to other more medically oriented ESM behaviors of managing medication, safety, and seizures. This was consistent with prior studies on ESM among patients with epilepsy [12,37].

MINDSET 2.0 has added features to screen for comorbidities of depression symptoms and subjective cognitive difficulties and to recommend training programs of UPLIFT and/or HOBSCOTCH, respectively. Patients with epilepsy have a greater prevalence of psychiatric comorbidities than the general population and are at greater risk of being undertreated for depression, which is consistently demonstrated to be inversely associated with successful ESM [41,42]. Consistent with previous studies, more than 33% (n=2) of MINDSET participants reported symptoms of depression. A question item regarding suicidal ideation is also embedded in the NDDI-E survey. Adding an alert in the AP specific to suicidality was suggested that will further highlight this issue. Specific reference to this item has since been included in the neurologist MINDSET 2.0 training.

People with epilepsy have an increased risk for impaired memory, executive function, attention, and social skills in comparison to people without epilepsy [43,44]. Cognitive function can be further compromised by early onset of epilepsy, frequent recurrent seizures, and polytherapy [43]. Low QOLIE-31 cognitive subscale scores (≤70%) were consistent with the prevalence of cognitive impairment of 60% and 70% for patients with epilepsy in general [43,45]. Patients with epilepsy often have cognitive impairments that can compromise their use of MINDSET 2.0. The positive ratings for many usability parameters are encouraging and consistent with previous studies of ESM interventions that report outcomes of increased awareness, feelings of autonomy, ESM adherence, and a sense of hope [13,17] and significant improvement in cognitive health and depression. Previous studies have also reported that between 30% and 50% of patients with epilepsy will underestimate or overestimate their cognitive performance, so objective assessments, in conjunction with caregiver reports as well as mood and anxiety measures, are required to establish truly comprehensive and accurate cognitive assessments [46,47]. MINDSET assists HCPs in identifying and referring those patients who require further objective neuropsychological assessment but does not provide a direct referral pathway for neuropsychological testing nor does it replace neuropsychological testing.

The words used in MINDSET were understandable, suggesting an acceptable reading level. Participants appreciated the program’s comprehensiveness while also indicating it was of appropriate duration, appraisals that are typically inversely associated. However, participants had difficulty when responding to and interpreting items that they perceived as incongruous to their experience. The “forced” selection resulted in inaccurate responses. The addition of a “not applicable” response option to previously published survey items and associated adjustment of the decision algorithm logic will help to increase response validity and increase the salience of AP recommendations. While not a majority opinion, the difficulties in AP development reinforced the advantage of involving caregivers in using MINDSET and generating the AP, and of ensuring HCPs reinforce it as well as instruct on its use.

### Strengths and Limitations

A large sample size for usability testing is not necessarily required since statistical significance is not a determining factor to establish usability problems, but a limited sample does reduce generalizability to the broader epilepsy population [48]. MINDSET 2.0 offers the potential for increased accessibility and reach. Paradoxically, there is a “digital divide” that may naturally limit reach to some patients. The study’s requirement of access to internet-accessible devices introduces a selection bias toward higher socioeconomic status and greater digital literacy. Further, the system supports Spanish, but testing in Spanish was limited (n=1), and thus, no conclusions about user experiences with the Spanish translation can be drawn, but additional testing of the Spanish version is indicated. Patients with more severe forms of epilepsy that impair their ability to use MINDSET were also excluded from this study, further limiting generalizability to the broader population of people with epilepsy. Therefore, future testing of MINDSET among more representative clinical groups is needed.

MINDSET elicits self-reported responses from patients with epilepsy and is therefore subject to associated recall bias. Consistent with their long-term experience with epilepsy, participants were adherent to prescribed ASD dosing and medication management behaviors. However, self-reported medication adherence has low sensitivity for unintentional nonadherence because people with epilepsy tend to overestimate their self-reported medication adherence [49,50] and unintentionally fail to notice suboptimal ASD adherence [51].

MINDSET 2.0 was developed to be used independently, and patients indicated that they could complete it unaided, but the study supported the benefits of enabling assistance from caregivers and/or clinic staff, especially accounting for patients who have comorbid cognitive challenges. Caregiver and family support is an important associated factor, so content addressing this was suggested for inclusion since the concept of “familias” is particularly salient to the Hispanic culture [7]. Inclusion of caregivers, as well as clinic staff, can improve the validity of data collection, goal selection, and AP management. Studies on the application of MINDSET 2.0 in conjunction with caregivers and community health workers are planned.

### Conclusion

This study indicates that MINDSET 2.0 may be a useful program for addressing low adherent ESM behaviors, providing tailored management intervention based on patient-mediated goals and strategy selection, screening for depression and memory function, identification of salient social determinants, and cuing linkage of patients to local resources designed to alleviate social needs and to evidence-based ESM epilepsy programs. Findings from this formative usability study replicated those from earlier versions of MINDSET but also highlighted the need for flexible protocols to enable caregiver support and lead to the initiation of enhancements to mitigate inaccurate algorithm decision logic and item response formatting prior to feasibility testing in neurology clinics [12,37].

## Supplementary material

10.2196/79540 Multimedia Appendix 1 MINDSET 2.0 usability instrument.

## Abbreviations

AP: action plan
ASD: anti-seizure drug
EFCST: Epilepsy Foundation Central & South Texas
ESM: epilepsy self-management
ESMS: Epilepsy Self-management Survey
HCP: health care provider
HOBSCOTCH: Home-Based Self-Management and Cognitive Training Changes Lives
MEW: Managing Epilepsy Well
MINDSET: Management Information & Decision Support Epilepsy Tool
MPH: Masters in Public Health
NDDI-E: Neurological Disorders Depression Inventory in Epilepsy
PACES: Program for Active Consumer Engagement in Self-Management
QoL: Quality of Life
QOLIE-10: Quality of Life in Epilepsy Inventory-10
QOLIE-31: Quality of Life in Epilepsy Inventory-31
UPLIFT: Using Practice and Learning to Increase Favorable Thoughts
