# Supplementary material for: An Online Epilepsy Self-Management Tool for Patients With Epilepsy: A Formative Usability Study
Source: JMIR Hum Factors. 2026 Aug 5;13:e79540. doi: 10.2196/79540 (PMC13440935; doi:10.2196/79540)
Supplement: Supplementary file 1 [file humanfactors-v13-e79540-s001.docx]

**MINDSET*PLUS* USABILITY SURVEY**

**© University of Arizona Department of Neurology and**

**University of Texas School of Public Health**

**2021**

**Instructions**

Please let us know what you think about MINDSET*Plus*. This survey is designed to find out what patients think about this computer-based program. We want to know what you like and what you think could be better.

Your participation is voluntary. You do not have to answer any questions that make you feel uncomfortable. It is YOUR CHOICE to answer the questions on this survey.

It is very important that you answer every question as truthfully as you can.

*The following questions ask about the* MINDSET*Plus program as a whole. This includes the My EPILEPSY, My GOALS and the My PLAN section.*

**SECTION I**

*Please circle the answer that best describes how you feel about your experience with* MINDSET*Plus.*

1. The time it took to use MINDSET*Plus* was________.

○ Too Quick	○ Just right	○ Too Long

2. The information I received from MINDSET*Plus* was__________.

○ Helpful	○ Not Helpful	○ Don’t know

3. The information I got from MINDSET*Plus* ________.

○ Can be Trusted	○ Can’t Be Trusted	○ Don’t know

4. The *questions* I was asked in MINDSETPlus will help me to think carefully about how I manage my epilepsy.
5. The information I received from MINDSETPlus will help me better manage my epilepsy.
   - Yes	○ No	○ Don't know
6. MINDSETPlus gave me most of the information I needed.
   - Yes	○ No	○ Don't know
7. Having MINDSETPlus suggest MEW Network program(s) was helpful.
   - Yes	○ No	○ Don't know
8. The MEW Network program(s) that MINDSETPlus suggested for me are appropriate for my needs.
   - Yes	○ No	○ Don't know
9. The information provided in MINDSET*Plus* will help me talk to my doctor or nurse about my epilepsy.
   - Yes	○ No	○ Don't know
10. I knew and understood most of the words used in MINDSET*Plus*.
    - Yes	○ No	○ Don't know
11. I needed help understanding the questions in MINDSETPlus
    - Yes	○ No	○ Don't know
12. How does MINDSET*Plus* compare to other seizure programs you have used in the clinic?

- Less useful
- As useful
- More useful
- I have never used other seizure programs

13. I would use MINDSET*Plus* in a clinic visit.
    - Yes	○ No	○ Don't know
14. I would recommend MINDSET*Plus* to other patients.
    - Yes	○ No	○ Don't know
15. The size of the letters made it easy to read.
    - Yes	○ No	○ Don't know
16. MINDSET*Plus* was easy to use.
    - Yes	○ No	○ Don't know
17. MINDSET*Plus* will help me achieve my epilepsy management goals.
    - Yes	○ No	○ Don't know
18. If you selected YES, please explain how MINDSETPlus helped you achieve your epilepsy management goals. If you selected NO, then please explain why not?

**SECTION II**

*Please circle the answer that best describes how you feel about:*

19. The **entire program**.

○ Like a Lot	○ Like	○ Dislike	○ Dislike a Lot	○ Not Sure

If you selected *“Dislike”* or *“Dislike a Lot”* please explain why:

20. The **colors** used in this program.

○ Like a Lot	○ Like	○ Dislike	○ Dislike a Lot	○ Not Sure

If you selected *“Dislike”* or *“Dislike a Lot”* please explain why:

21. The **buttons** used in this program.

○ Like a Lot	○ Like	○ Dislike	○ Dislike a Lot	○ Not Sure

If you selected *“Dislike”* or *“Dislike a Lot”* please explain why:

22. The **hand-held device** used for this program.

○ Like a Lot	○ Like	○ Dislike	○ Dislike a Lot	○ Not Sure

If you selected *“Dislike”* or *“Dislike a Lot”* please explain why:

23. The **size of the letters**.

○ Like a Lot	○ Like	○ Dislike	○ Dislike a Lot	○ Not Sure

If you selected *“Dislike”* or *“Dislike a Lot”* please explain why:

24. The **layout of the screens**.

○ Like a Lot	○ Like	○ Dislike	○ Dislike a Lot	○ Not Sure

If you selected that you *“Dislike”* or *“Dislike a Lot”* the layout of the screens. Please explain why:

**SECTION III**

*Please tell us how easy it was to perform each of the following activities:*

25. Entering your responses into MINDSET:

○ Not easy	○ Somewhat easy	○ Very Easy	○ Don’t Know

26. Understanding the instructions for “My Goals”:

○ Not easy	○ Somewhat easy	○ Very Easy	○ Don’t Know

27. Choosing a topic to work on:

○ Not easy	○ Somewhat easy	○ Very Easy	○ Don’t Know

28. Choosing your strategies:

○ Not easy	○ Somewhat easy	○ Very Easy	○ Don’t Know

29. Understanding the Action Plan:

○ Not easy	○ Somewhat easy	○ Very Easy	○ Don’t Know

**SECTION IV**

*Please tell us how easy it was to perform each of the following activities:*

30. Downloading the PDF version of the Action Plan

○ Not easy	○ Somewhat easy	○ Very Easy	○ Don’t Know

31. Understanding the PDF version of the Action Plan

○ Not easy	○ Somewhat easy	○ Very Easy	○ Don’t Know

32. Understanding the layout of the PDF version of the Action Plan

○ Not easy	○ Somewhat easy	○ Very Easy	○ Don’t Know

33. Understanding the content of the PDF version of the Action Plan

○ Not easy	○ Somewhat easy	○ Very Easy	○ Don’t Know
